# Polidocanol versus glucose in the treatment of telangiectasia of the lower limbs (PG3T): Protocol for a randomized, controlled clinical trial

**DOI:** 10.1097/MD.0000000000004812

**Published:** 2016-09-30

**Authors:** Matheus Bertanha, Paula Angeleli Bueno de Camargo, Regina Moura, Winston Bonetti Yoshida, Rafael Elias Farres Pimenta, Jamil Victor de Oliveira Mariúba, Giovana Piteri Alcantara, Dênia Reis de Paula, Marcone Lima Sobreira

**Affiliations:** a Discipline of Angiology and Vascular of Departament of Surgery and Orthopedics, Botucatu Medical School, UNESP; b Botucatu Medical School, UNESP, Botucatu, São Paulo, Brazil.

**Keywords:** Glucose solution, Hypertonic, Sclerosing solutions, Sclerotherapy, Spider veins, Telangiectasis, Varicose veins, Veins

## Abstract

**Background:**

Telangiectasias are defined as small venules abnormally dilated, located in the intradermal portion of the skin, of reddish or bluish tinge, their diameter not exceeding 1 mm; they are classified by the American Venous Forum as mild venous disease CEAP C1. Conventional treatment consists of chemical sclerotherapy, considered a minimally invasive technique with rapid clinical recovery. A wide variety of sclerosing solutions can be used for this purpose.

**Methods/design:**

This project intends to include 96 patients that will be randomized to a triple-blind study. Inclusion criteria are women between 18 and 65 years, with telangiectasia on the lateral thigh. Male patients, female patients with chronic venous disease CEAP 2 to 6, women with allergies, pregnant, breastfeeding, with any type of skin problems or any decompensated clinical disease will be excluded. All patients included will be submitted to venous ultrasound mapping in order to rule out venous disease not clinically visible, deep venous system insufficiency, and insufficiency of the ostial valve of the great saphenous vein. One group will be treated with glucose 75% solution and the other will receive polidocanol 0.2% diluted in glucose 70%. Each patient will receive only 1 treatment session in 1 single member. The volume of sclerosing solution will not exceed 5 mL and the treatment area will be limited to a region of 150 cm^2^ on the lateral thigh. Clinical follow-up will be: 1 initial visit, when the clinical report will be filled; photographic record and treatment with sclerotherapy (D0); follow-up visits after 7 and 60 days (D7 and D60, respectively), always with clinical and photographic documentation.

**Discussion:**

The project intends to evaluate the efficacy and safety of sclerotherapy in eliminating telangiectasia in a predetermined area in order to establish efficacy and safety parameters for the treatments presented.

**Conclusion:**

This protocol for clinical trial will provide date to determine the efficacy and safety of sclerotherapy with the solutions presented.

**Trial registration identifier:**

ClinicalTrial.gov NCT02657252 Date: 01/12/2016 (retrospectively registered).

## Abstract 2

Supplemental Digital Content is available in the text

## 1 Background

Telangiectasias are defined as abnormally dilated venules (or capillaries) in the intradermic portion of the skin, with reddish or bluish tinge, their diameter not exceeding 1 mm.^[1]^ Usually they do not cause clinical symptoms, but rather complaints about cosmetic; some patients, however, report some pain, itching, cramping or heaviness at the site.^[1]^ They have been classified by the American Venous Forum as mild venous disease C1 CEAP (clinical, etiologic, anatomic, and pathophysiologic classification—CEAP), along with the reticular veins, where no varicose veins are found.^[2,3]^

Treatment of telangiectasia is supported by some researchers as the factor of clinical and cosmetic improvement for patients.^[1,4,5]^ The techniques that have been most widely used are those promoting sclerosis of the target veins, using sclerosing intravenous solutions or transdermal laser.^[6]^ The use of laser promotes the elimination of telangiectasia by selective transdermal photothermolysis, obliterating the vessel with a heat-induced injury provoked by a specific light length;^[6]^ chemical sclerotherapy uses a variety of chemical agents that primarily promote irritation, dehydration, and destruction of the endothelial cells, resulting in elimination of the vein.^[7]^ There is no evidence, however, of more efficacy when comparing the chemical techniques to laser therapy.^[8]^ Chemical sclerotherapy is the most widely used method due to its technical simplicity, rapid return to daily activities, and low cost. It is usually well tolerated by patients since it is almost painless, requiring no anesthesia, the discomfort being related to the venipuncture and drug infusion.^[7,9]^ All sclerosing agents available on the market have been reported as effective in the treatment of cosmetic venous disease, the choice being guided by the preference and experience of the attending physician.

Hypertonic glucose is a potent hyperosmolar sclerosing solution that promotes dehydration and destruction of endothelial cells and obliteration of the vessel lumen. Polidocanol is a detergent that acts by destroying the vein wall lipids and the intercellular “cement,” what causes endothelial maceration and obliterates the vessel.

Some papers point out that the sclerosing power of polidocanol and sodium tetradecyl sulfate (STS) would be higher than hyperosmolar agents;^[10–12]^ however, both are more fluid and can flow to unwanted vessels. Those opposed to the use of polidocanol, warn against adverse effects such as tissue necrosis, allergic reactions, anaphylaxis, increased risk of deep venous thrombosis (DVT), pulmonary embolism (PE), scotoma, and gas embolization (when used in foam form).^[13]^ These events are rare and often related to miscalculated doses.^[14,15]^ Some minor adverse events such as hyperpigmented spots, as well as mild systemic adverse events such as coughing, brief scotoma, light lipothymia, are described as being more often associated with the use of polidocanol.^[16]^ On the other hand, other authors believe that the side effects can be avoided if the volume and infusion time are well calculated, therefore rendering better results without a higher rate of adverse events.^[17]^

A commercial presentation of polidocanol 0.2% diluted in glucose 70% has been used in Brazil with apparent success and safety but with no scientific proof. In a pilot study of this institution, there was a trend toward better results with the use of this solution, justifying, therefore, what we intend to test in this study, comparing the solution of polidocanol 0.2% diluted in glucose 70% to glucose 75% solution for the treatment of lower limbs telangiectasia.

The hypothesis to be tested is that the polidocanol 0.2% diluted in glucose 70% is more efficient than glucose 75% in eliminating lower limbs telangiectasia (efficacy). Differences in safety will also be investigated (minor and major adverse events).

## 2 Methods/design

### 2.1 Study design

Single-center, prospective, randomized, triple-blind study to compare the efficacy in promoting the disappearance of telangiectasia in a treated lower extremity comparing photos taken before treatment and 60 days post-treatment. Two commonly used sclerosing solutions (polidocanol 0.2% diluted in glucose 70%, and glucose 75% solution) will be used and compared. Safety of the solutions will also be investigated, evaluating any major (DVT and skin necrosis) and minor adverse events (hyperpigmented spots, small ulcers and edema at the site of the application). The sample size was statistically estimated as 96 treated limbs (see Statistical analysis).

### 2.2 Ethics approval and consent to participate

This study will be conducted in accordance to the principles of the Declaration of Helsinki, ISO14155, Data Protection Act, and guidelines for Good Clinical Practice. The Research Ethics Committee (REC) of the Botucatu Medical School, UNESP, São Paulo, Brazil, has approved this study, which was registered under the number 4127–2012 (see REC, supplemental digital content 1). During the screening visit, all patients will be fully informed of the benefits and risks of the study and the principal investigator will obtain the signature of the free and informed consent form (ICF) for all participants, before enrollment (see ICF, supplemental digital content 2). The subjects may leave this study at any point in time without any constraint, whenever they will.

All data will be sent to the ethics committee in the end of the study or if occur any major adverse effect. As the study provides a unique intervention, you can not interrupt the treatment.

The ClinicalTrials.gov identifier for this study is NCT02657252, obtained on January 12, 2016 (Acronym PG3T).

### 2.3 Selection, inclusion, randomization, and masking

The sample will be of convenience, starting with a random and temporal list of patients who wish to do aesthetic treatment with sclerotherapy in the lower extremities due to mild venous disease - CEAP C1, in our institution. The patients will be invited by telephone to participate in the study, following the order of established by consulting the electronic database of our institution. They will be invited to attend a scheduled outpatient visit for clinical evaluation, and in order to be informed about the working methods. In this screening evaluation, patients who present telangiectasia on the lateral side of the thigh will then be invited to participate in the study and sign the ICF.

The inclusion will be random, using a spreadsheet electronically generated by the web-based free-access computer program Stat Trek (http://stattrek.com/Tables/Random.aspx). The patients will be randomized in 2 groups; one will receive treatment with 0.2% polidocanol diluted in glucose 70%; the other will be treated with glucose 75%. A trained nurse will generate and store all data in an opaque envelope for the entire duration of the study and prepare the solutions that will be used in the treatments, with sequential allocation in accordance with the order of addition. The solutions will be prepared in a medication room (different from the treatment room); the product (5 mL) will be drawn a sterile syringe identified only with the patient's protocol number, a few minutes before use, in such a way that the physician who will perform the procedure will have no contact with this phase of the research. It is noteworthy that the 2 drugs are colorless and odorless, with similar viscosity, what will promote an efficient blinding. Thus, all study participants will be blinded, patients, care providers, outcome assessors, and data analysts (triple blinded).

### 2.4 Eligibility criteria

The inclusion and exclusion criteria are depicted in Table 1.

**Table 1:**
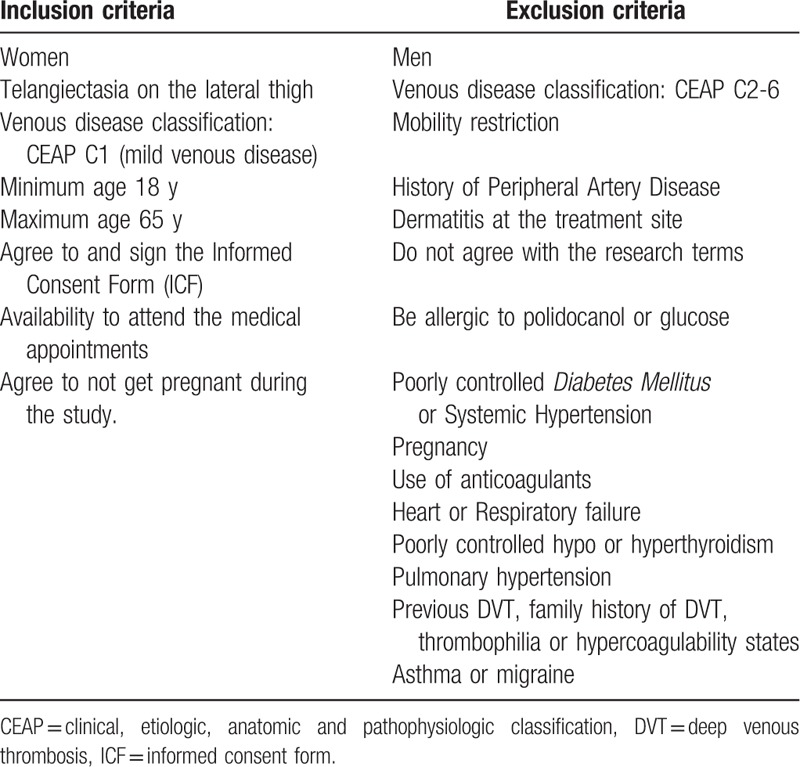
Eligibility criteria.

### 2.5 Pretreatment details

The patients will be included if they present telangiectasia not related to varicose veins on the side of the thigh of one of the lower extremities. The clinical diagnosis will be confirmed before treatment by duplex ultrasonography. Clinical data such as comorbidities, demographics and other relevant information will be compiled in specific forms for further analysis. All patients who do not meet the inclusion criteria during the screening evaluation will be referred to the routine outpatient clinic.

### 2.6 Treatment area

**Figure 1:**
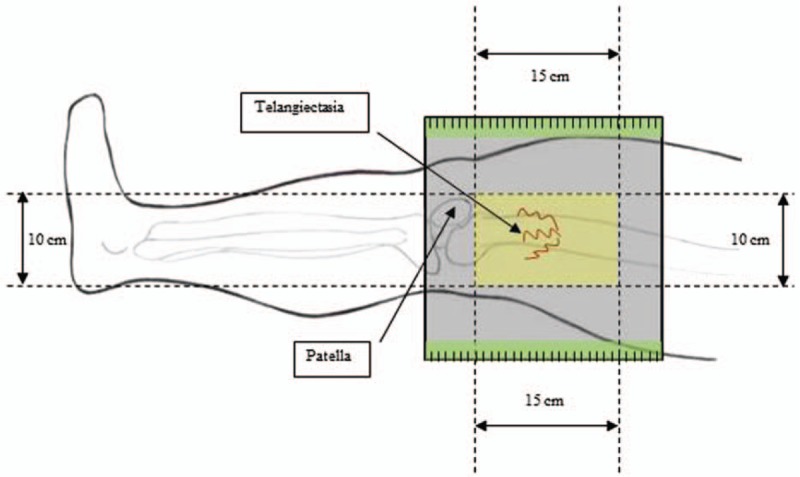
Schematic representation of the treatment area in this study.

The treatment area was defined as the lateral thigh of a lower limb (limited to 1 member per patient, independent of the side) and the target area is a rectangle 15 cm long and 10 cm wide, generating a 150 cm^2^ treatment area. For better reproducibility of the results, a template was made of cloth with velcro fixation to be positioned on the line of the knee joint. The open rectangular area will therefore be positioned 5 cm above the knee joint and the lower and anterior vertex of the rectangle will be positioned on the lateral aspect of the patella (Fig. 1). In order to facilitate the analysis of the pictures, a plastic ruler will be displayed at the edges of the template. After photographing the target area, small dots of permanent marker will be made in order to define the target area; the template will then be removed to facilitate the treatment. Telangiectasias that cross the edges of the template will also be entirely treated, so that small variations in the position of the template will not cause difficulties for the analysis of the results.

### 2.7 Photographic record

Photos of the patients will be taken in order to register the 3 stages of the research: one before the treatment (D0), one 7 days after treatment (D7), and the last 60 days after treatment (D60). A high definition camera will be used to take all the pictures (D7000 Nikon Lens™: AF-S Nikkor 18–105 mm ™ 1: 3.5–5.6G). In order to avoid possible bias, comparisons will always be conducted on the same patient through photographic analysis before and after treatment, to establish disappearance of telangiectasias on the treated area (see “Outcomes”), as well as the occurrence of hyperpigmented spots. The pictures will always be taken in the same position, with the patient lying supine on a stretcher, with the photographic template in place (see “treatment area”), with the target area facing the camera that will be at a distance of 60 cm from the patient. The environment will be controlled to maintain optimum light level. The pictures will be stored in files for further analysis using the web-based free-access software ImageJ. Each picture will be identified only with each patient's protocol number and time it was taken (D0—pre-treatment, D7—7 days after treatment; D60 - 60 days after treatment). The ImageJ Software will be used to measure the length of the telangiectasia treated, residual telangiectasias, and hyperpigmented spots. The results obtained in pixels will be transformed by a simple mathematical rule of three by conversion in the ruler of the photographic template. By this mean we will obtain objective measurements of both the disappearance of telangiectasias and of any patches of hyperpigmentation. Personal information and photos will not be published, except for group results and the parts that could not identify the person.

### 2.8 Treatment (D0)

After the photographic record of the first visit (D0), all patients will be treated similarly, aiming to eliminate all telangiectasia of the target area. The same physician will perform all the procedures. The treatment technique will be the conventional one, consisting of careful direct puncture of the telangiectasias in order to avoid infiltration of the subcutaneous tissue. The procedure will be conducted until the telangiectasias are undetectable. The maximum volume allowed by puncture will not exceed 0.3 mL. A plastic syringe, luer lock tip, with 3 mL will be used to apply the solution (BD), and the needle will be 13 × 0.4 mm (Terumo - or 27G ^1/2^ inch). The punctures will be occluded with a small cotton ball and a square piece of microporus surgical tape.

**Figure 2:**
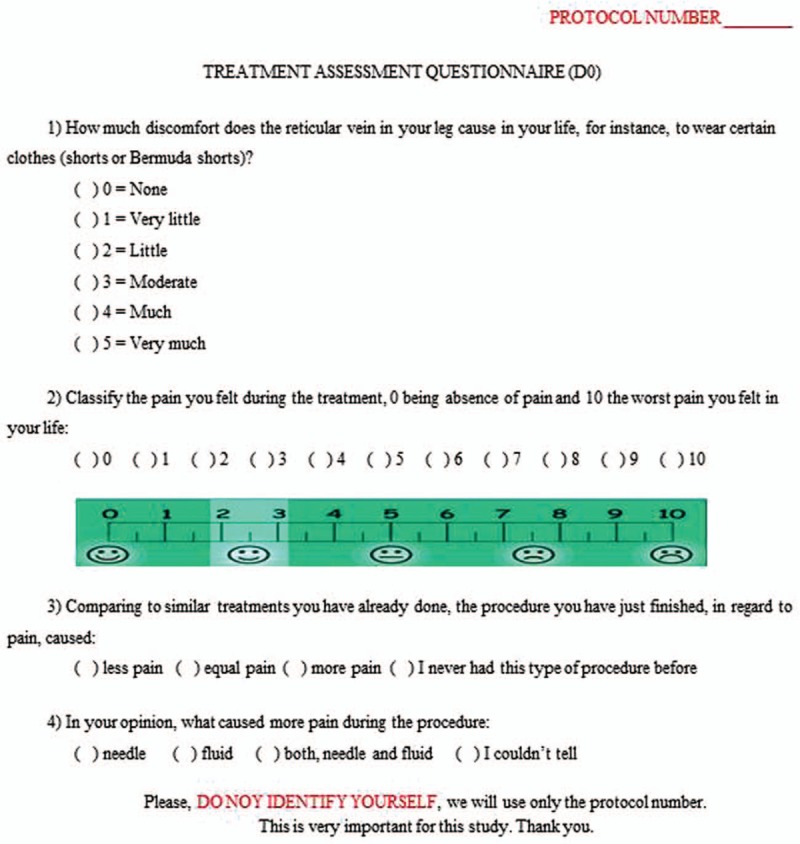
Assessment form to be filled by the patient after the treatment.

All events during the treatment will be recorded in this visit, including the amount of agent used for the disappearance of telangiectasias, possible allergic reactions, and others. Soon after the session, the patient will be invited to answer a questionnaire with questions related to discomfort or pain triggered by the treatment, that was already used in a previous similar study (Fig. 2).^[18]^

**Figure 3:**
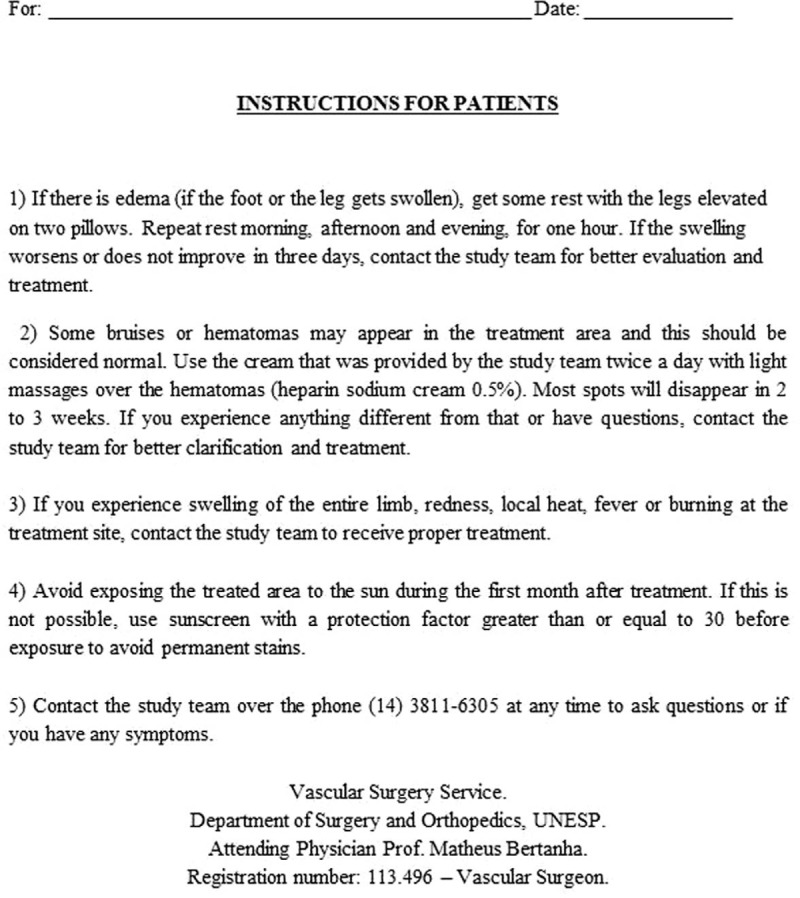
Instructions sheet for patients after the sclerotherapy treatment for telangiectasia.

Patients will receive verbal and written instructions on how to proceed with post-treatment care (Fig. 3). All patients will receive a tube of cream containing sodium heparin 0.5% to treat bruises, hematomas, or any phlebitis resulting from the treatment. The medication should be applied for topical use twice daily for 2 weeks.

### 2.9 Post-treatment (D7)

Around the seventh day, considering the patient's availability to attend, a new clinical evaluation will be carried out (7 days with a tolerance of ± 4 days after the treatment session; D7). On this visit, the initial results of the treatment will be evaluated, with main focus on minor adverse events such as mild allergies, localized edema, cough, lipothymia, brief scotoma, bruises, and phlebitis, as well as major adverse events such as syncope, DVT, severe allergies, or other major injuries to health. If identified, these events will be recorded in a specific form for this visit. Early phlebitis of the treated vessels can occur with some frequency, and if present will be drained through punctures with a needle (13 gauge × 4.5 BD) and extraction of the thrombus with gentle compression. During this visit, pictures will be taken in the manner described above and the images stored for future analysis.

### 2.10 Post-treatment (D60)

After 60 days of treatment, with ± 15-day, the last clinical evaluation will be conducted. This visit is intended to solve issues relating to the efficacy and safety of the treatment, and a new photographic record will be held under the same conditions previously described. In addition, any delayed adverse events will be recorded (such as allergies, edema, respiratory symptoms, syncope, scotoma, suspected DVT, phlebitis, and ulcerations). In case of late phlebitis, treatment will be the same as described for “D7.”

If the patient did not attend any of the visits will be carried out consecutive daily telephone calls to try to rescue the patient and schedule a new visit, provided it remains within the deadline.

**Figure 4:**
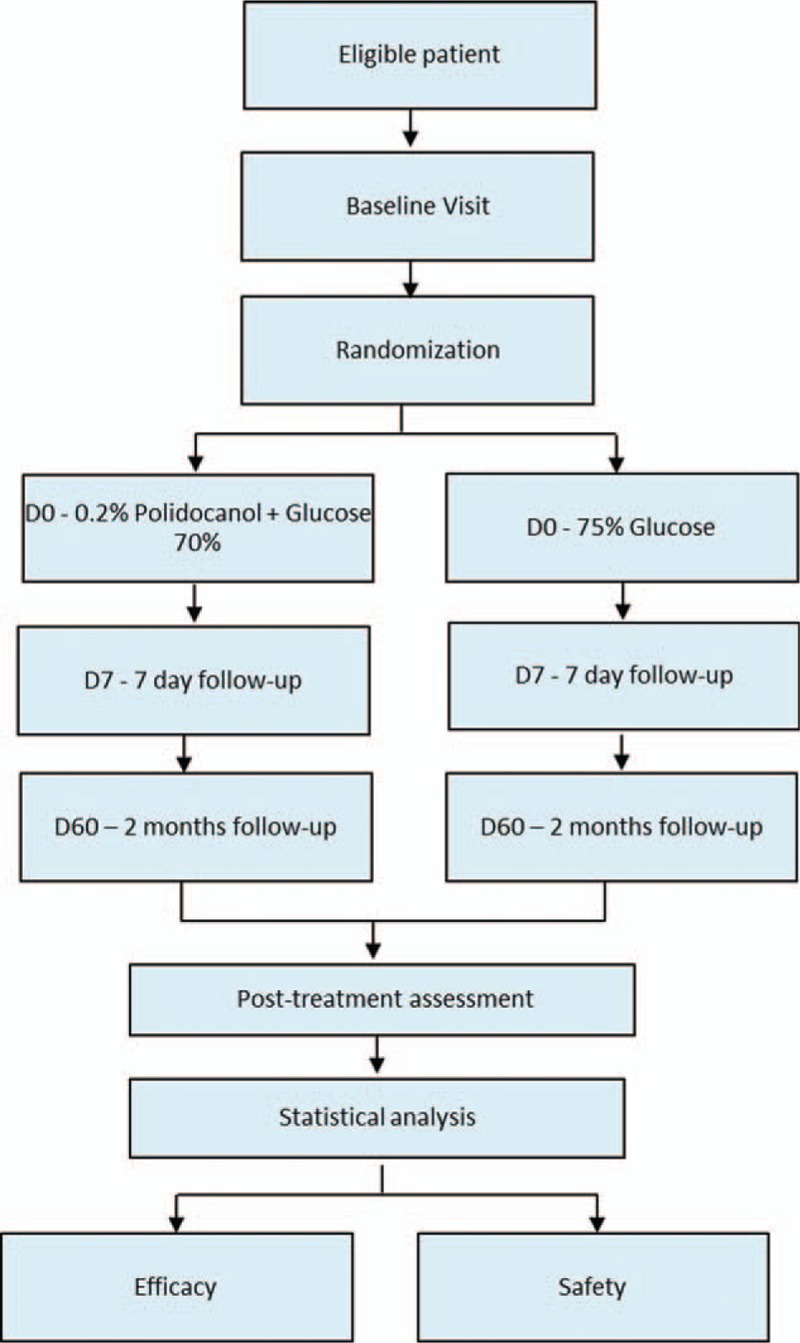
Flowchart of patients in the study.

To summarize the stages of the study, see flow diagram in Fig. 4 and see the supplemental digital content 3 with Standard Protocol Items: Recommendations for Interventional Trials (SPIRIT).

### 2.11 Outcomes

#### 2.11.1 Efficacy endpoint

The primary objective (efficacy) will be based on the ability of each treatment to promote the disappearance of treated telangiectasia after 60 days. The analysis will be done by comparing the initial lengths of the telangiectasia before treatment (D0) and the residual telangiectasia after 60 days of treatment (D60), always limiting the comparison to the same patient and same treatment area as previously described. The linear measurement tool of the ImageJ software will measure the length in pixels and transform by a rule of three, through the scale ruler, rendering the results comparable for numerical equivalence in centimeters. Each case will be examined by 2 external evaluators (2 vascular surgeons with experience in the topic), unaware of the treatments conducted, the study therefore being triple-blind. According to the image, the examiners will produce absolute values that will be statistically compared.

For possible conflicting results where very different values are displayed (>10% difference), a meeting will be held between the examiners and a consensus will be established (still blinded to the study treatments). Finally, for statistical purposes, the average of the results obtained for all cases by the 2 examiners will be used.

#### 2.11.2 Safety endpoint

The hyperpigmented spots are relatively expected events for this type of treatment and will be assessed by objective measurement in the same manner as previously described in the section “Efficacy Outcome.” Still as primary objective, safety, hyperpigmented spots will be objectively measured using the ImageJ software, and the comparison will be with the length of the telangiectasias before the treatment. The same examiners will make these analyzes as previously described.

#### 2.11.3 Secondary outcomes

All the situations that may be indirectly involved with the results in terms of efficacy and safety, among them volume of drug used, number of punctures, skin color, and pain related to the treatment, will be analyzed in the secondary endpoints. Other adverse events as mild allergies, localized or diffuse edema, cough, mild lipothymia, hematomas and phlebitis, besides major adverse events such as syncope, DVT, severe allergies or other major health injuries will also be evaluated, as secondary endpoints.

### 2.12 Statistical analysis

Based on alpha = 0.05, and assuming a standard deviation in the primary endpoint score of 1.5, with 48 participants in each arm, we have >80% power to detect a minimum difference in scores of 0.9. Only 1 extremity will be included per patient and the analysis will be conducted comparing results per patients. It is expected that 10–20% of patients will be lost during follow-up. To promote the most statistically rigorous method, we will use a larger sample size of at least 115 patients. Data will be evaluated either by Student's *t* test or nonparametric Mann–Whitney test (if relevant conditions are not met). Categorical variables (e.g., proportions) will be compared between groups using the χ 2 or Fisher's exact test when necessary. Scores between the groups will be compared with Mann–Whitney test. Multiple comparisons between and within multinomial populations will be analyzed by Goodman association test. All statistical analyzes will be conducted using STATA v11 software (Stata Corp, College Station, TX). All analyses will be performed on an intent-to-treat basis.

### 2.13 Adverse effects expected

The solutions selected for the study (polidocanol 0.2% diluted in glucose 70%, and glucose 75% solution) are widely used regionally and throughout the world as sclerosing agents in sclerotherapy procedures for telangiectasia. The products, techniques, concentrations, and dilutions are as many as possible. There are reports of adverse events, mostly considered mild, rarely presented with the use of glucose 75% solution; nevertheless, polidocanol has been associated in rare occasions with serious adverse events and a slightly higher frequency of mild adverse events, mostly related to incorrect use or volume employed. The pain caused, hematomas, local or diffuse edema, spots of hyperpigmentation or depigmentation, phlebitis, and small ulcers are adverse events considered minor and are the most frequently documented events after this kind of treatment.^[16]^ All adverse events will be recorded and the adverse events considered as serious will be reported to the REC.

### 2.14 Protocol amendments

Any amendments to the protocol and information provided to participants will be submitted to the REC for approval prior to implementation. Substantial amendments may only be implement after REC has been obtained, whereas nonsubstantial amendments can be implemented without written approval for REC. Data and source documents will be stored in such a way that can be accessed at a later date for the purposes of monitoring or inspection by the REC. After the end of the study, the results from the trial will be submitted for publication in a peer-reviewed journal, following CONSORT 2010 guidelines. Authorship of presentations and reports related will be in the name of the collaborative group.

## 3 Discussion

Several studies have demonstrated that chemical sclerotherapy of telangiectasia of the lower extremities is a common and effective treatment. A wide range of solutions has been employed for this purpose.^[16,19,20]^ Virtually all studies show that the several solutions used are effective in promoting the disappearance of the vessels.^[7]^ Nevertheless, few scientific papers present results of randomized, well-controlled trials,^[10,11,16]^ and we found no study comparing the effects of Polidocanol 0.2% diluted in glucose 70% to glucose 75%.

The main objective of this study is to present reliable results comparing the selected sclerosing solutions. It is intended to demonstrate the efficacy in promoting the disappearance of telangiectasias of the treated area and the safety of the use of these agents, observing the frequencies of major and minor adverse events, especially hyperpigmented spots.

## Supplementary Material

Supplemental Digital Content
