# Supplementary material for: Polidocanol versus glucose in the treatment of telangiectasia of the lower limbs (PG3T): Protocol for a randomized, controlled clinical trial
Source: Medicine (Baltimore). 2016 Sep 30;95(39):e4812. doi: 10.1097/MD.0000000000004812 (PMC5265902; doi:10.1097/MD.0000000000004812)
Supplement: Supplemental Digital Content [file medi-95-e4812-s001.pdf]

unesp
Universidade Estadual Paulista
Faculdade de Medicina de Botucatu

Distrito Rubião Junior, s/nº - Botucatu – S.P.
CEP: 18.618-970
Fone/Fax: (0xx14) 3811-6143
e-mail secretaria: capellup@fmb.unesp.br
e-mail coordenadoria: tsarden@fmb.unesp.br

Ética
Comitê de Ética em Pesquisa
Fls. nº 46

Registrado no Ministério da Saúde
em 30 de abril de 1997

Botucatu, 05 de março de 2012 Of. 56/2012

Ilustríssimo Senhor
Prof. Titular Winston B. Yoshida
Departamento de Cirurgia e Ortopedia da
Faculdade de Medicina de Botucatu

Prezado Dr. Winston,

De ordem do Senhor Coordenador deste CEP, informo que o Projeto de Pesquisa **(Protocolo CEP 4127-2012)** **"Estudo clínico randomizado e duplo cego comparando dois métodos de escleroterapia para veias reticulares e telangiectasias em membros inferiores"**, a ser conduzido por Matheus Bertanha, orientado por Vossa Senhoria, com a colaboração de Marcone Lima Sobreira, Fernando Cordeiro Pimentel, Hamilton de Almeida Rollo e Regina Moura, recebeu do relator **parecer favorável**, aprovado em reunião de 05/03/2012.

Situação do Projeto: ***APROVADO***. *Os pesquisadores deverão apresentar ao CEP ao final da execução do Projeto o* **"Relatório Final de Atividades"**.

Atenciosamente,

Alberto Santos Capelluppi
Secretário do CEP

UNIVERSIDADE ESTADUAL PAULISTA
"JÚLIO DE MESQUITA FILHO"
Campus de Botucatu

Comitê de Ética em Pesquisa
Fls. nº 47

Botucatu, 20 de março de 2014.

Ilmo Sr.
Prof.Dr. Trajano Sardenberg
Coordenador do Comitê de Ética em Pesquisa.

Venho por meio desta, solicitar a alteração do orientador da pesquisa **(Protocolo CEP 4127-2012) "Estudo clínico randomizado e duplo cego comparando dois métodos de escleroterapia para veias reticulares e telangiectasias em membros inferiores"** que foi registrada no nome do Dr. Winston Bonetti Yoshida e será agora orientada pelo Dr. Marcone Lima Sobreira. Solicito ainda que este estudo passe a ser considerado como título acadêmico para defesa de Doutorado do orientado Matheus Bertanha. Estas mudanças são necessárias para contemplar o atual momento de desenvolvimento desta pesquisa e para atender os interesses de todos os envolvidos.

Agradecemos desde já.
Sem mais para o momento.
Atenciosamente.

Prof. Dr. Winston Bonetti Yoshida
Ex-Orientador

Prof. Dr. Marcone Lima Sobreira
Novo Orientador

Dr. Matheus Bertanha
Orientado

Autorizo 27/03/14
Prof. Dr. Trajano Sardenberg
Coordenador do CEP

Faculdade de Medicina de Botucatu - Departamento de Cirurgia e Ortopedia
Distrito de Rubião Júnior, s/n CEP 18618-970 Botucatu São Paulo Brasil
Tel /Fax 55 14 3811 6269

TERMO DE CONSENTIMENTO LIVRE E ESCLARECIDO
(Resolução CFM n. 1.931/2009 – Código de Ética Médica)

Por meio deste instrumento particular, eu abaixo assinado (a), procurei a Equipe de Cirurgia Vascular do HC UNESP Botucatu, cujo responsável neste caso é o Dr. Matheus Bertanha, para o tratamento de minhas varizes e por isso autorizo a realização deste procedimento, estando ciente das seguintes condições:

Eu, ________________________________ RG ___________ RG Hospitalar _____________, estou sendo convidado a participar de um estudo denominado "AVALIAÇÃO ENTRE DOIS MÉTODOS DE ESCLEROTERAPIA PARA VEIAS RETICULARES E TELANGIECTASIAS EM MEMBROS INFERIORES" cujo objetivo é a melhora do quadro de varizes. A justificativa desse estudo é que não se sabe qual dos dois tratamentos é o melhor para a minha doença e a minha participação neste estudo é receber o tratamento com um desses medicamentos para secar vasinhos (escleroterapia): Glicose ou Polidocanol diluído em glicose. Isso será realizado em uma faixa do membro inferior direito (   ) / esquerdo (   ) em apenas uma sessão de aplicação em veias selecionadas para melhor atender o objetivo do estudo e será necessário que você volte em dois retornos para acompanhamento do procedimento. Depois dessa fase, o tratamento será completado de convencional no setor de Cirurgia Ambulatorial, sem necessidade de novos retornos.

Fui orientada que, ao participar da pesquisa terei alguns benefícios, tais como a melhoria de algumas varizes da perna, mas por outro lado, fui alertada e devidamente esclarecida de possíveis desconfortos e riscos decorrentes do estudo, levando-se em conta que é uma pesquisa, e os resultados positivos ou negativos somente serão obtidos após a sua realização. Assim, são possíveis complicações a não melhora estética, ocorrência de manchas na área tratada, feridas, hematomas, dor, flebites, trombose, embolias entre outras. De qualquer forma, já sabemos que estas complicações não acontecem muito, o que justifica a realização do trabalho.

Estou ciente de que minha privacidade será respeitada, ou seja, meu nome ou qualquer outro dado ou elemento que possa, de qualquer forma, me identificar, será mantido em sigilo.

Também fui informado de que posso me recusar a participar do estudo, ou retirar meu consentimento a qualquer momento, sem precisar justificar, e de, por desejar sair da pesquisa, não sofrerei qualquer prejuízo à assistência que venho recebendo. Foi-me esclarecido, igualmente, que eu posso optar pelo método alternativo que é a cirurgia de varizes.

Os pesquisadores envolvidos com o referido projeto são Matheus Bertanha, Marcone Lima Sobreira, Regina Moura, Winston Bonetti Yoshida e Hamilton Almeida Rollo, todos médicos da Cirurgia Vascular do Hospital das Clínicas da Faculdade de Medicina de Botucatu, e com eles poderei manter contato pelo telefone (14) 3811-6269/3811-6305.

É assegurada a assistência médica durante toda pesquisa, bem como me é garantido o livre acesso a todas as informações e esclarecimentos adicionais sobre o estudo e suas conseqüências, enfim, tudo o que eu queira saber antes, durante e depois da minha participação, exceto pelo tipo de medicamento utilizado, que só será revelado no fim da pesquisa.

Enfim, tendo sido orientado quanto ao teor de todo o aqui mencionado e compreendido a natureza e o objetivo do já referido estudo, manifesto meu livre consentimento em participar, estando totalmente ciente de que não há nenhum valor econômico, a receber ou a pagar, por minha participação.

Caso ocorra algum dano decorrente da minha participação no estudo, serei devidamente acompanhado neste hospital visando a minha recuperação, estando esta pesquisa dentro do que determina a lei.

Botucatu, ____/_________/20_____. Assinatura____

Nome:____RG:_______________

____

Dr. Matheus Bertanha – CRM 113496
Médico Responsável pelo Ambulatório de Escleroterapia.

SPIRIT 2013 Checklist: Recommended items to address in a clinical trial protocol and related documents*

Section/item	Item No	Description	Addressed on page number
**Administrative information**			
Title	1	Descriptive title identifying the study design, population, interventions, and, if applicable, trial acronym	______1,2,5___
Trial registration	2a	Trial identifier and registry name. If not yet registered, name of intended registry	______2,5_____
	2b	All items from the World Health Organization Trial Registration Data Set	______ 5_____
Protocol version	3	Date and version identifier	_______2_____
Funding	4	Sources and types of financial, material, and other support	______14_____
Roles and responsibilities	5a	Names, affiliations, and roles of protocol contributors	_____1,13____
	5b	Name and contact information for the trial sponsor	______NA_____
	5c	Role of study sponsor and funders, if any, in study design; collection, management, analysis, and interpretation of data; writing of the report; and the decision to submit the report for publication, including whether they will have ultimate authority over any of these activities	______NA_____
	5d	Composition, roles, and responsibilities of the coordinating centre, steering committee, endpoint adjudication committee, data management team, and other individuals or groups overseeing the trial, if applicable (see Item 21a for data monitoring committee)	______NA_____

**Introduction**			
Background and rationale	6a	Description of research question and justification for undertaking the trial, including summary of relevant studies (published and unpublished) examining benefits and harms for each intervention	______3,4____
	6b	Explanation for choice of comparators	______3,4____
Objectives	7	Specific objectives or hypotheses	______4_____
Trial design	8	Description of trial design including type of trial (eg, parallel group, crossover, factorial, single group), allocation ratio, and framework (eg, superiority, equivalence, noninferiority, exploratory)	______5_____
**Methods: Participants, interventions, and outcomes**			
Study setting	9	Description of study settings (eg, community clinic, academic hospital) and list of countries where data will be collected. Reference to where list of study sites can be obtained	______5,6____
Eligibility criteria	10	Inclusion and exclusion criteria for participants. If applicable, eligibility criteria for study centres and individuals who will perform the interventions (eg, surgeons, psychotherapists)	__6, table 1 _
Interventions	11a	Interventions for each group with sufficient detail to allow replication, including how and when they will be administered	______7,8____
	11b	Criteria for discontinuing or modifying allocated interventions for a given trial participant (eg, drug dose change in response to harms, participant request, or improving/worsening disease)	______NA_____
	11c	Strategies to improve adherence to intervention protocols, and any procedures for monitoring adherence (eg, drug tablet return, laboratory tests)	______9,10____
	11d	Relevant concomitant care and interventions that are permitted or prohibited during the trial	___ Figure 3___
Outcomes	12	Primary, secondary, and other outcomes, including the specific measurement variable (eg, systolic blood pressure), analysis metric (eg, change from baseline, final value, time to event), method of aggregation (eg, median, proportion), and time point for each outcome. Explanation of the clinical relevance of chosen efficacy and harm outcomes is strongly recommended	_____10,11____
Participant timeline	13	Time schedule of enrolment, interventions (including any run-ins and washouts), assessments, and visits for participants. A schematic diagram is highly recommended (see Figure 4)	______5-10____

Sample size	14	Estimated number of participants needed to achieve study objectives and how it was determined, including clinical and statistical assumptions supporting any sample size calculations	______11_____
Recruitment	15	Strategies for achieving adequate participant enrolment to reach target sample size	______11_____

Methods: Assignment of interventions (for controlled trials)

Allocation:			
Sequence generation	16a	Method of generating the allocation sequence (eg, computer-generated random numbers), and list of any factors for stratification. To reduce predictability of a random sequence, details of any planned restriction (eg, blocking) should be provided in a separate document that is unavailable to those who enrol participants or assign interventions	______5,6____
Allocation concealment mechanism	16b	Mechanism of implementing the allocation sequence (eg, central telephone; sequentially numbered, opaque, sealed envelopes), describing any steps to conceal the sequence until interventions are assigned	______6______
Implementation	16c	Who will generate the allocation sequence, who will enrol participants, and who will assign participants to interventions	______6______
Blinding (masking)	17a	Who will be blinded after assignment to interventions (eg, trial participants, care providers, outcome assessors, data analysts), and how	______6______
	17b	If blinded, circumstances under which unblinding is permissible, and procedure for revealing a participant's allocated intervention during the trial	______NA_____

Methods: Data collection, management, and analysis

Data collection methods	18a	Plans for assessment and collection of outcome, baseline, and other trial data, including any related processes to promote data quality (eg, duplicate measurements, training of assessors) and a description of study instruments (eg, questionnaires, laboratory tests) along with their reliability and validity, if known. Reference to where data collection forms can be found, if not in the protocol	____6-11____
	18b	Plans to promote participant retention and complete follow-up, including list of any outcome data to be collected for participants who discontinue or deviate from intervention protocols	____6-11___

Data management	19	Plans for data entry, coding, security, and storage, including any related processes to promote data quality (eg, double data entry; range checks for data values). Reference to where details of data management procedures can be found, if not in the protocol	_____6-11____
Statistical methods	20a	Statistical methods for analysing primary and secondary outcomes. Reference to where other details of the statistical analysis plan can be found, if not in the protocol	______11_____
	20b	Methods for any additional analyses (eg, subgroup and adjusted analyses)	______11_____
	20c	Definition of analysis population relating to protocol non-adherence (eg, as randomised analysis), and any statistical methods to handle missing data (eg, multiple imputation)	______11_____
**Methods: Monitoring**			
Data monitoring	21a	Composition of data monitoring committee (DMC); summary of its role and reporting structure; statement of whether it is independent from the sponsor and competing interests; and reference to where further details about its charter can be found, if not in the protocol. Alternatively, an explanation of why a DMC is not needed	_____6,12____
	21b	Description of any interim analyses and stopping guidelines, including who will have access to these interim results and make the final decision to terminate the trial	______6______
Harms	22	Plans for collecting, assessing, reporting, and managing solicited and spontaneously reported adverse events and other unintended effects of trial interventions or trial conduct	______6______
Auditing	23	Frequency and procedures for auditing trial conduct, if any, and whether the process will be independent from investigators and the sponsor	_____NA_____
**Ethics and dissemination**			
Research ethics approval	24	Plans for seeking research ethics committee/institutional review board (REC/IRB) approval	_____NA_____
Protocol amendments	25	Plans for communicating important protocol modifications (eg, changes to eligibility criteria, outcomes, analyses) to relevant parties (eg, investigators, REC/IRBs, trial participants, trial registries, journals, regulators)	_____13______

Consent or assent	26a	Who will obtain informed consent or assent from potential trial participants or authorised surrogates, and how (see Item 32)	______5_______
	26b	Additional consent provisions for collection and use of participant data and biological specimens in ancillary studies, if applicable	______NA_____
Confidentiality	27	How personal information about potential and enrolled participants will be collected, shared, and maintained in order to protect confidentiality before, during, and after the trial	______5,8______
Declaration of interests	28	Financial and other competing interests for principal investigators for the overall trial and each study site	______14_____
Access to data	29	Statement of who will have access to the final trial dataset, and disclosure of contractual agreements that limit such access for investigators	______13_____
Ancillary and post-trial care	30	Provisions, if any, for ancillary and post-trial care, and for compensation to those who suffer harm from trial participation	______NA____
Dissemination policy	31a	Plans for investigators and sponsor to communicate trial results to participants, healthcare professionals, the public, and other relevant groups (eg, via publication, reporting in results databases, or other data sharing arrangements), including any publication restrictions	______13_____
	31b	Authorship eligibility guidelines and any intended use of professional writers	______13_____
	31c	Plans, if any, for granting public access to the full protocol, participant-level dataset, and statistical code	______13_____
**Appendices**			
Informed consent materials	32	Model consent form and other related documentation given to participants and authorised surrogates	Additional file 2
Biological specimens	33	Plans for collection, laboratory evaluation, and storage of biological specimens for genetic or molecular analysis in the current trial and for future use in ancillary studies, if applicable	_____NA______

*It is strongly recommended that this checklist be read in conjunction with the SPIRIT 2013 Explanation & Elaboration for important clarification on the items. Amendments to the protocol should be tracked and dated. The SPIRIT checklist is copyrighted by the SPIRIT Group under the Creative Commons "Attribution-NonCommercial-NoDerivs 3.0 Unported" license.
